# Forced Sexual Initiation and Early Sexual Debut and Associated Risk Factors and Health Problems Among Adolescent Girls and Young Women — Violence Against Children and Youth Surveys, Nine PEPFAR Countries, 2007–2018

**DOI:** 10.15585/mmwr.mm7047a2

**Published:** 2021-11-26

**Authors:** Ashleigh L. Howard, Sherri Pals, Brianna Walker, Regina Benevides, Greta M. Massetti, Rose Patricia Oluoch, Obinna Ogbanufe, Louis Herns Marcelin, Toni Cela, Chabila C. Mapoma, Elizabeth Gonese, Wezi Msungama, Daniel Magesa, Alick Kayange, Katelyn Galloway, Rose Apondi, Lydia Wasula, Owen Mugurungi, Getrude Ncube, Iven Sikanyiti, Justin Hamela, Gerald V. Kihwele, Nozipho Nzuza-Motsa, Janet Saul, Pragna Patel

**Affiliations:** ^1^Strategic Innovative Solutions, LLC, Clearwater, Florida; ^2^Division of Global HIV and TB, Center for Global Health, CDC; ^3^Division of Violence Prevention, National Center for Injury Prevention and Control, CDC; ^4^CDC Kenya; ^5^CDC Nigeria; ^6^University of Miami, Coral Gables, Florida; ^7^Interuniversity Institute for Research and Development, Port-au-Prince, Haiti; ^8^Department of Population Studies, University of Zambia, Harare, Zimbabwe; ^9^CDC Zimbabwe; ^10^CDC Malawi; ^11^CDC Tanzania; ^12^Walter Reed Army Institute of Research, U.S. Department of Defense, Tanzania; ^13^CDC Eswatini; ^14^CDC Uganda; ^15^Uganda Ministry of Gender, Labour and Social Development, Kampala, Uganda; ^16^Zimbabwe Ministry of Health and Child Care, Harare, Zimbabwe; ^17^Zambia Central Statistics Office, Lusaka, Zambia; ^18^Malawi Ministry of Gender, Children, Disability and Social Welfare, Lilongwe, Malawi; ^19^Tanzania Ministry of Health, Community Development, Gender, Elderly and Children, Dar es Salaam, Tanzania; ^20^Eswatini Ministry of Health, Mbabane, Eswatini; ^21^Office of the Global AIDS Coordinator, U.S. Department of State.

Adolescent girls and young women aged 13–24 years are disproportionately affected by HIV in sub-Saharan Africa (*1*), resulting from biologic, behavioral, and structural* factors, including violence. Girls in sub-Saharan Africa also experience sexual violence at higher rates than do boys (*2*), and women who experience intimate partner violence have 1.3–2.0 times the odds of acquiring HIV infection, compared with those who do not (*3*). Violence Against Children and Youth Survey (VACS) data during 2007–2018 from nine countries funded by the U.S. President’s Emergency Plan for AIDS Relief (PEPFAR) were analyzed to estimate prevalence and assess factors associated with early sexual debut and forced sexual initiation.^†^ Among adolescent girls and young women aged 13–24 years who ever had sex, the prevalence of lifetime sexual violence ranged from 12.5% to 49.3%, and forced sexual initiation ranged from 14.7% to 38.9%; early sexual debut among adolescent girls and young women aged 16–24 years ranged from 14.4% to 40.1%. In multiple logistic regression models, forced sexual initiation was associated with being unmarried, violence victimization, risky sexual behaviors, sexually transmitted infections (STIs), and poor mental health. Early sexual debut was associated with lower education, marriage, ever witnessing parental intimate partner violence during childhood, risky sexual behaviors, poor mental health, and less HIV testing. Comprehensive violence and HIV prevention programming is needed to delay sexual debut and protect adolescent girls and young women from forced sex.

VACS are nationally representative, multistage cluster household surveys of persons aged 13–24 years. All study protocols included oral informed consent, parental consent for minors, safeguards to protect privacy and confidentiality of participants, and referrals to postviolence care as needed.^§^ CDC and in-country Institutional Review Boards approved study protocols.^⁋^ This report examines lifetime experiences of sexual violence, early sexual debut, and forced sexual initiation among adolescent girls and young women aged 13–24 years in Eswatini,** Haiti, Kenya, Malawi, Nigeria, Tanzania, Uganda, Zambia, and Zimbabwe, using 2007–2018 data from VACS.^††^ Lifetime sexual violence included ever experiencing 1) unwanted sexual touching, 2) unwanted attempted sex, 3) pressured or coerced sex, or 4) physically forced sex. Early sexual debut was defined as first sexual intercourse at or before age 15 years among adolescent girls and young women aged 16–24 years who had ever had sex with or without violence. Forced sexual initiation was defined as pressured, coerced, or physically forced first sex among adolescent girls or young women aged 13–24 years who had ever had sex. Orphan status was defined as having one or both parents deceased before the 18th birthday. Weighted prevalences of lifetime sexual violence, early sexual debut, and forced sexual initiation were estimated for each country using SAS (version 9.4; SAS Institute); prevalences were weighted to the most recent census or population projections to account for the multistage cluster design and nonresponse. Multiple logistic regression models were fit to the data combined across all countries to examine the odds of forced sexual initiation and early sexual debut by demographic characteristics and childhood experiences and health problems and behaviors. These models included a fixed effect for country and accounted for survey design, including stratification and clustering, and were adjusted to control for age and marital status.

Prevalence of lifetime sexual violence among adolescent girls and young women varied across countries, ranging from 12.5% (Zimbabwe) to 49.3% (Eswatini) (Table 1). Among those who had ever had sex, the prevalence of forced sexual initiation ranged from 14.7% (Zimbabwe) to 38.9% (Malawi) and early sexual debut ranged from 14.4% (Zimbabwe) to 40.1% (Nigeria). Among adolescent girls and young women who had ever had sex, the odds of having experienced forced sexual initiation were elevated among those aged 13–15 years (adjusted odds ratio [aOR] = 1.77), those who experienced nonpenetrative sexual violence during childhood (aOR = 2.38) and who experienced emotional violence during childhood (aOR = 1.44) (Table 2). Odds were lower among those who were ever married or cohabitating (aOR = 0.47) and those who never attended school (aOR = 0.56). The odds of early sexual debut were higher among adolescent girls and young women aged 16–19 years (aOR = 3.29), those who had no education (aOR = 5.16) or less than primary education (aOR = 2.19), were ever married or cohabitating (aOR = 3.03), or who had witnessed parental intimate partner violence before age 18 years (aOR = 1.31). The odds of forced sexual initiation or early sexual debut did not differ by orphan status, childhood physical violence, or witnessing violence in the community during childhood.

**TABLE 1:** Prevalence of sexual violence, forced sexual initiation, and early sexual debut among adolescent girls and young women aged 13–24 years — Violence Against Children and Youth Surveys, nine countries, 2007–2018

Country (survey yr)	Lifetime sexual violence*		Forced sexual initiation^†^		Early sexual debut^§^	
	No.	Weighted prevalence,^¶^ % (95% CI)	No.	Weighted prevalence,^¶^ % (95% CI)	No.	Weighted prevalence,^¶^ % (95% CI)
Eswatini** (2007)	1,232	49.3 (45.0–53.6)	646	19.5 (15.1–23.9)	620	16.8 (13.2–20.4)
Haiti (2013)	1,438	40.3 (36.7–43.8)	733	23.5 (19.9–27.0)	682	32.6 (28.4–36.8)
Kenya (2018)	1,335	25.5(22.5–28.5)	520	23.1 (18.6–27.6)	471	18.3 (14.0–22.6)
Malawi (2013)	1,028	33.6 (28.8–38.4)	595	38.9 (32.2–45.7)	557	32.4 (26.9–38.0)
Nigeria (2014)	1,737	35.6 (32.4–38.8)	882	25.0 (21.0–29.0)	807	40.1 (34.7–45.5)
Tanzania (2009)	1,947	35.3 (30.5–40.0)	261	33.4 (25.4–41.5)	219	21.9 (12.8–31.0)
Uganda (2015)	3,143	44.3 (40.8–47.9)	1,867	18.8 (15.4–22.2)	1,784	25.0 (20.4–29.5)
Zambia (2014)	880	31.7 (28.2–35.2)	515	27.6 (23.0–32.1)	469	27.6 (22.5–32.7)
Zimbabwe (2017)	7,893	12.5 (11.6–13.3)	3,462	14.7 (13.4–16.0)	3,434	14.4 (13.1–15.7)

**Abbreviation:** VACS = Violence Against Children and Youth Surveys.

* Lifetime sexual violence is defined as unwanted touching, unwanted attempted sex, pressured or coerced sex, or physically forced sex. For this analysis, lifetime sexual violence was examined for adolescent girls and young women aged ≤24 years.

^†^ Forced sexual initiation defined as first sexual intercourse was physically forced, pressured, or coerced, among adolescent girls and young women aged 13–24 years who had ever had sex. Definitions of pressured sex varied among countries.

^§^ Early sexual debut defined as first sex at aged ≤15 years among adolescent girls and young women aged 16–24 years who had ever had sex.

^¶^ Weights accounted for the multistage cluster sampling design (e.g., enumeration area, household, and household member), nonresponse, and calibration to a known population (i.e., the most recent census for Eswatini, Kenya, Tanzania, Uganda, and Zimbabwe; for the remaining countries, the country’s population projections for the year of VACS data used).

** Formerly Swaziland.

**TABLE 2:** Prevalence of and odds ratios for forced sexual initiation and early sexual debut among adolescent girls and young women aged 13–24 years who had ever had sex,* (N = 20,770), by characteristics — Violence Against Children and Youth Surveys, nine countries, 2007–2018

Characteristic/Health risk behavior	Forced or sexual initiation^†^		Early sexual debut^§^	
	Weighted average prevalence^¶^ (95% CI)	aOR (95% CI)	Weighted average prevalence^¶^(95% CI)	aOR (95% CI)
Age group at time of survey, yrs				
13–15	38.1 (30.6–46.2)	1.8** (1.3–2.5)	—^§^	—^§^
16–19	27.4 (24.2–30.9)	1.2 (0.9–1.5)	36.3 (32.7–40.0)	3.3** (2.6–4.2)
20–24	21.5 (19.4–23.7)	Referent	18.4 (16.5–20.5)	Referent
Education				
None	13.0 (9.3–17.9)	0.6** (0.4–0.8)	59.4 (51.1–67.2)	5.2** (3.6–7.4)
Less than primary	29.2 (22.8–36.6)	1.1 (0.7–1.8)	36.7 (29.3–44.6)	2.2** (1.5–3.2)
Primary or higher	24.5 (22.5–26.6)	Referent	19.5 (17.6–21.6)	Referent
Orphan status^††^				
No	24.1 (22.1–26.1)	Referent	24.0 (21.9–26.2)	Referent
One parent	26.1 (22.1–30.5)	1.1 (0.8–1.5)	24.6 (21.1–28.4)	1.0 (0.8–1.3)
Both parents	18.8 (13.0–26.2)	0.8 (0.5–1.3)	27.8 (20.4–36.7)	1.4 (0.9–2.1)
Ever married or lived as married				
Yes	18.6 (16.5–20.9)	0.5** (0.4–0.6)	29.5 (27.1–32.1)	3.0^¶^ (2.3–3.9)
No	34.4 (30.8–38.2)	Referent	15.9 (13.3–19)	Referent
Any nonpenetrative sexual violence during childhood^§§^				
Yes	40.6 (36.1–45.3)	2.4** (1.8–3.1)	27.2 (22.8–32.1)	1.3 (1.0–1.6)
No	19.6 (17.8–21.6)	Referent	24.5 (22.4–26.6)	Referent
Any childhood physical violence^¶¶^				
Yes	27.2 (24.7–29.9)	1.2 (0.9–1.5)	22.9 (20–26)	0.8 (0.6–1.1)
No	21.7 (19.1–24.5)	Referent	27.0 (24.2–30.1)	Referent
Any emotional violence during childhood***				
Yes	31.1 (27.5–35.1)	1.4** (1.1–1.8)	28.7 (24.4–33.4)	1.3 (1.0–1.7)
No	22.3 (20.4–24.3)	Referent	23.6 (21.6–25.8)	Referent
Ever witnessed parental intimate partner violence during childhood^†††^				
Yes	29.0 (25.2–33.1)	1.3 (1.0–1.6)	32.8 (28.9–36.9)	1.3** (1.0–1.7)
No	24.7 (22.1–27.5)	Referent	25.0 (22.1–28.2)	Referent
Witnessed violence in the community during childhood^§§§^				
Yes	29.1 (25.9–32.6)	1.3 (1.0–1.6)	28.1 (24.5–31.9)	1.2 (0.9–1.5)
No	23.6 (20.6–26.9)	Referent	27.7 (24.7–31)	Referent

**Abbreviation:** aOR = adjusted odds ratio.

* Sex was defined as ever experiencing vaginal, oral, or anal sex (Kenya, Malawi, Nigeria, Uganda, Zambia, and Zimbabwe); vaginal or anal intercourse (Haiti); or sexual intercourse (Eswatini and Tanzania).

^†^ Forced sexual initiation was defined as having sexual intercourse at first sexual encounter through physical force, pressure, or coercion, among adolescent girls and young women aged 13–24 years who had ever had sex. Definitions of pressured sex varied among countries.

^§^ Early sexual debut was defined as first sex at age ≤15 years among adolescent girls and young women aged 16–24 years who had ever had sex. Participants aged 13–15 years were excluded from this analysis because they are still in the period during which early sexual debut could occur.

^¶^ Prevalence is weighted to the population of each of the nine included countries (except where fewer countries are included, as noted).

** Statistically significant (p<0.05).

^††^ Orphan status was defined as having one or both parents deceased before the 18th birthday.

^§§^ Nonpenetrative sexual violence was defined as unwanted sexual touching or unwanted attempted sex.

^¶¶^ Physical violence included being “punched, kicked, whipped, or beat with an object,” “choked, smothered, drowned, or burned,” or “threatened with a weapon” by an intimate/romantic partner, peer, family member or caregiver, or adult in community before age 18 years.

*** Emotional violence included having a parent, caregiver, or other adult telling child they were not loved, do not deserve to be loved, that they wished the child was dead or never born, or if they ever ridiculed or put down the child before age 18 years.

^†††^ Ever witnessed parental intimate partner violence included seeing or hearing a parent punched, kicked, or beaten by their partner. Data from Eswatini, Haiti, Tanzania, and Zimbabwe were not included.

^§§§^ Ever witnessed violence in the community included seeing anyone get attacked outside of home or family environment. Data from Eswatini, Haiti, Tanzania, and Zimbabwe were not included.

Compared with adolescent girls and young women who did not experience forced sexual initiation, those who did had elevated odds of engaging in transactional sex^§§^ during the past 12 months (aOR = 1.6), infrequent condom use during the past 12 months (among those who were never married) (aOR = 1.7), ever having had an STI (aOR = 1.6), experiencing one or more (aOR = 2.8) and two or more (aOR = 2.6) types of violence during the past 12 months, having recent moderate or severe mental distress (aOR = 1.6), and ever having suicidal thoughts (aOR = 2.1) (Table 3). Compared with those who did not experience early sexual debut, those who did had increased odds of having multiple sexual partners during the past 12 months (aOR = 1.7), infrequent condom use among those who were ever married (aOR = 2.1) or never married (aOR = 1.9), having recent moderate or severe mental distress (aOR = 1.5), and ever having attempted suicide (among those who ever thought of suicide) (aOR = 2.1). The odds were lower for ever having been tested for HIV (aOR = 0.4) and having been tested during the past year (aOR = 0.5). The odds of alcohol abuse did not differ among those who did and did not experience forced sexual initiation or early sexual debut.

**TABLE 3:** Prevalence of health outcomes and behaviors among adolescent girls and young women aged 13–24 years who had ever had sex,* and their association with forced sexual initiation and early sexual debut (N = 20,770) — Violence Against Children and Youth Surveys, nine countries, 2007–2018

Health outcome and behavior	Forced sexual initiation^†^			Early sexual debut^§^		
	Weighted average prevalence^¶^		aOR (95% CI)	Weighted average prevalence^¶^		aOR (95% CI)
	Yes, % (95% CI)	No, % (95% CI)		Yes, % (95% CI)	No, % (95% CI)	
Risky sexual behavior (past 12 mos)**						
Transactional sex	8.7 (6.6–11.4)	5.2 (4.1–6.5)	1.6^††^ (1.0–2.3)	6.6 (4.9–8.8)	5.3 (4.2–6.6)	1.3 (0.8–2.0)
Multiple sex partners	9.1 (6.2–13.3)	6.0 (4.7–7.5)	1.4 (0.9–2.3)	7.9 (5.6–11.1)	6.1 (4.6–8.1)	1.7^††^ (1.1–2.8)
**Infrequent condom use (past 12 mos)**						
Ever married or cohabiting females	95.9 (92.5–97.7)	96.6 (95.6–97.4)	0.8 (0.4–1.5)	97.8 (96.3–98.6)	95.3 (93.7–96.4)	2.1^††^ (1.1–3.7)
Never married females	71.9 (63.4–79.0)	59.0 (53.1–64.7)	1.7^††^ (1.2–2.6)	74.4 (66.7–80.8)	57.4 (51.4–63.2)	1.9^††^ (1.2–3.0)
**STI (lifetime)^§§^**	21.7 (17.5–26.6)	15.1 (13.4–17.0)	1.6^††^ (1.1–2.2)	14.7 (12.0–17.9)	17.4 (15.5–19.5)	1.0 (0.7–1.4)
**Recent violence (past 12 mos)^¶¶^**						
One or more types of violence	58.0 (52.6–63.2)	30.2 (28.0–32.4)	2.8^††^ (2.1–3.6)	36.3 (31.8–41.0)	36.1 (33.9–38.4)	1.1 (0.9–1.4)
Two or more types of violence	22.6 (19.4–26.2)	8.5 (7.3–9.7)	2.6^††^ (1.9–3.4)	11.5 (9.0–14.6)	11.1 (9.8–12.5)	1.1 (0.8–1.5)
Mental health problems***						
Moderate or severe mental distress in the past 30 days	58.9 (53.9–63.7)	46.4 (44.0–48.9)	1.6^††^ (1.3–2.1)	55.6 (50.7–60.3)	47.5 (44.9–50.1)	1.5^††^ (1.2–2.0)
Ever thought of suicide	24.8 (20.6–29.5)	12.7 (11.1–14.5)	2.1^††^ (1.6–3.0)	16.3 (12.7–20.6)	15.7 (13.9–17.7)	1.1 (0.8–1.5)
Ever attempted suicide among those who ever thought of suicide	32.9 (24.6–42.4)	23.6 (17.9–30.4)	1.7 (1.0–2.8)	39.5 (29.4–50.7)	21.9 (16.9–28.0)	2.1^††^ (1.2–3.7)
**Alcohol abuse in the past 30 days** ^†††^	7.6 (5.7–10.2)	9.3 (8.1–10.8)	0.8 (0.5–1.1)	6.8 (5.2–8.9)	9.2 (8.0–10.5)	0.8 (0.6–1.1)
**HIV testing**						
Ever tested for HIV	73.4 (68.3–77.9)	73.0 (70.5–75.3)	1.2 (0.9–1.7)	61.3 (56.0–66.3)	80.7 (78.4–82.8)	0.4^††^ (0.3–0.6)
HIV test in last 12 months	44.3 (38.7–50.0)	43.2 (40.4–46.0)	1.2 (0.9–1.5)	32.0 (27.9–36.5)	49.2 (46.6–51.9)	0.5^††^ (0.4–0.7)

**Abbreviations:** aOR = adjusted odds ratio; STI = sexually transmitted infection.

* Sex was defined as ever experiencing vaginal, oral, or anal sex (Kenya, Malawi, Nigeria, Uganda, Zambia, and Zimbabwe); vaginal or anal intercourse (Haiti); or sexual intercourse (Eswatini and Tanzania).

^†^ Forced sexual initiation was defined as having sexual intercourse at first sexual encounter through physical force, pressure, or coercion, among women and girls aged 13–24 years who had ever had sex. Definitions of pressured sex varied among countries.

^§^ Early sexual debut was defined as first sex at age ≤15 years among adolescent girls and young women aged 16–24 years who had ever had sex.

^¶^ Prevalence is weighted to the population of each of the nine included countries (except where fewer countries are included, as noted).

** Among adolescent girls and young women who had sex in the past 12 months. Data for Eswatini (risky sexual behavior) and Tanzania (multiple sex partners) were not included. Models for transactional sex and infrequent condom use did not include a fixed effect for country as it made the model unstable.

^††^ Statistically significant (p<0.05).

^§§^ STI was defined as ever having symptoms or a diagnosis of an STI. Data for Tanzania were not included. Model does not include a fixed effect for country because it made the model unstable.

^¶¶^ Data for Eswatini were not included.

*** Mental distress was defined as a score of five or higher on the Kessler 6 scale (https://onlinelibrary.wiley.com/doi/10.1002/mpr.310). Data for Eswatini and Tanzania were not included.

^†††^ Data for Eswatini and Tanzania were not included.

## Discussion

Sexual violence, forced sexual initiation, and early sexual debut were common among adolescent girls and young women in the nine countries examined. Increased odds of risky sexual behaviors among adolescent girls and young women who experienced forced sexual initiation and early sexual debut suggest that those with negative early sexual experiences are at increased risk for HIV acquisition. Forced sexual initiation was associated with having experienced violence during childhood and multiple types of recent violence, highlighting the complex interplay between early sexual experiences and violence. Association of forced sexual initiation and early sexual debut with recent mental distress, as well as with lifetime suicidal ideation and attempted suicide, indicate the deep and lasting impact of early or forced first sex on mental health.

These findings are consistent with previous studies showing that sexual violence, early sexual debut, and forced sexual initiation are associated with HIV acquisition and risky sexual behaviors (*4*,*5*). These findings also corroborate previous work relating recent violence with infrequent condom use and poor mental health (*6*) and underscore the need to provide postviolence care and mental health services for adolescent girls and young women to lower HIV risk (*7*). In these analyses, adolescent girls and young women who experienced early sexual debut had lower HIV testing rates despite higher HIV risk behaviors, indicating a need to reach girls who have early sexual debut for HIV testing services. These findings reinforce the importance of primary prevention of sexual violence and delayed sexual initiation as essential elements of HIV prevention and control (*8*).

The high prevalence of forced sexual initiation among the youngest members of this population who ever had sex demonstrates a need for sexual violence prevention programs to include girls aged <13 years. The following subgroups should be targeted for sexual violence prevention: in-school and never-married adolescent girls and young women, those who experienced sexual violence or emotional violence in childhood or forced sex, and families of adolescent girls and young women and their communities. In addition to encouraging girls to stay in school, programs might target adolescent girls and young women with primary education or less and those out of school, at risk for early marriage, and who witnessed parental intimate partner violence in childhood with programs to delay early sexual debut. Programs can adopt approaches from INSPIRE’s seven strategies for ending violence against children: implementation and enforcement of laws, norms and values, safe environments, parent and caregiver support, income and economic strengthening, response and support services, and education and life skills (*9*).

The findings in this report are subject to at least four limitations. First, data were self-reported, and are subject to recall bias. Second, slight variations in questions could contribute to differences in estimates across countries. Third, older data might not reflect recent changes in policies or programs. Finally, VACS only includes adolescent girls and young women living in households, so findings are not generalizable to those living in institutions or dormitories or to street youth.

Prevention of sexual violence and early sexual debut are key components of the comprehensive efforts of PEPFAR to control the HIV epidemic. In countries examined in this report, PEPFAR’s Determined, Resilient, AIDS-free, Mentored, and Safe (DREAMS) partnership provides a core package of interventions to help prevent sexual violence and HIV acquisition among adolescent girls and young women. These interventions include community mobilization and norms change, school-based interventions, caregiver programs, social protection, social asset building, economic strengthening, sexual and reproductive health care including postviolence care, and access to preexposure prophylaxis (*10*). HIV prevention among girls aged 9–14 years includes evidence-based programs to prevent violence and risky sexual behaviors and strengthen family and community support (*10*). Although some reductions in new HIV infections among adolescent girls and young women have been achieved, HIV incidence among this population remains high compared with that in young men (*1*) and calls for increased efforts to protect this vulnerable population with multiple evidence-based approaches to HIV and violence prevention.

**Summary**

**What is already known about this topic?**

Early sexual debut (first intercourse at or before age 15 years) and forced sexual initiation are associated with increased risk behaviors for HIV acquisition, including transactional sex and multiple sexual partners, but studies are limited.

**What is added by this report?**

During 2007–2018, in nine countries funded by the U.S. President’s Emergency Plan for AIDS Relief, 14.4% to 40.1% of adolescent girls and young women had early sexual debut. Among those with early sexual debut, the odds of having been tested for HIV was 40% lower and the odds of increased risky sexual behaviors were elevated.

**What are the implications for public health practice?**

Comprehensive violence and HIV prevention programs for adolescent girls and young women are needed to prevent early sexual debut and reduce the risk for HIV infection.

## Footnotes

* Structural factors include gender-based violence, child sexual abuse, orphanhood, low education level, spousal separation, harmful gender and societal norms, gender inequity, and unequal power.

† Detailed information about individual country survey partners, methodology, sampling design, samples, and response rates is available in VACS country reports. https://www.cdc.gov/violenceprevention/childabuseandneglect/vacs/reports.html

§
https://www.cdc.gov/violenceprevention/pdf/vacs/VACS-trainingwhitepaper.pdf

** Formerly Swaziland.

†† Eswatini: 2007, Tanzania: 2009, Haiti: 2013, Malawi: 2013, Nigeria: 2014, Zambia: 2014, Uganda: 2015, Zimbabwe: 2017, and Kenya: 2018.

§§ Sex in exchange for money, favors, or material support.
